# Factors Affecting the Manufacturing Industry Transformation and Upgrading: A Case Study of Guangdong–Hong Kong–Macao Greater Bay Area

**DOI:** 10.3390/ijerph18137157

**Published:** 2021-07-04

**Authors:** Fan Yang, Yanming Sun, Yuan Zhang, Tao Wang

**Affiliations:** 1 School of Management, Guangzhou University, Guangzhou 510000, China; 2112065012@e.gzhu.edu.cn (F.Y.); 201620567@gzcc.edu.cn (Y.Z.); 2 Research Center for High Quality Development of Modern Industry, Guangzhou University, Guangzhou 510000, China; 3 School of Management, Guizhou University of Commerce, Guiyang 550014, China; 4 Department of Building Surveying, Faculty of Built Environment, University of Malaya, Kuala Lumpur 50603, Malaysia; 17221416@siswa.um.edu.my

**Keywords:** manufacturing industry, transformation and upgrading, synergetics theory

## Abstract

This study aims to analyze the development trend of the manufacturing industry transformation and upgrading in the Guangdong–Hong Kong–Macao Greater Bay Area (2008–2018). On the basis of synergetics, the order parameter method of factor analysis is used to study these factors. The results show that: (1) There are five slow variable factors, such as intelligent manufacturing industry, technological innovation, scale agglomeration, market demand, and fixed asset investment, which are important power sources of the transformation and upgrading of the manufacturing industry in Greater Bay Area. The development of these factors is relatively mature, and they cooperate with each other. (2) Similar to a fast variable of manufacturing development ecology, green development is an important coordinating factor in removing bottlenecks. Finally, suggestions for the development of the transformation and upgrading of the manufacturing industry are put forward.

## 1. Introduction

The manufacturing industry is a key element of high-quality economic development [1], its productivity growth potential is higher than that of other industries [2]. Through its own advantages, such as capital accumulation [3], scale economy [4], and technological progress [5], it can produce greater spillover effects on other industries [6]. However, with the implementation of the “re-industrialization” strategy of developed countries in Europe and the United States, China’s manufacturing industry is facing greater challenges. Therefore, it is particularly important for China to realize the transformation and upgrading of the manufacturing industry (TUMI).

In recent years, TUMI has become the focus of academic attention. (1) Some scholars study the influencing factors of TUMI. For example, Li et al. [7] analyzed the factors affecting the transformation and upgrading of the logistics industry in Ningbo. Taking Thailand’s automobile and hard disk industry as an example, Kohpaiboon et al. [8] studied the role of the international production network and industrial cluster in the process of upgrading. Dou et al. [9] evaluated the manufacturing competitiveness of G20 countries and analyzed the effect of factors on manufacturing competitiveness based on the diamond theory. (2) Some scholars have explored the path of TUMI. For example, Zhao et al. [10] put forward the path of Internet economy transformation and upgrading of the metallurgical industry in Inner Mongolia. Ye [11] analyzed the reasons that hinder further adjustment and upgrading of China’s industrial structure. (3) Some scholars study the countermeasures of the regional manufacturing industry. For example, Huang et al. [12] put forward the strategy of TUMI in Taizhou. Liu et al. [13] believed that TUMI could be promoted by integrating global innovation resources. Dou et al. [14] used the entropy clustering method to analyze the spatial pattern of manufacturing development. Loren and Eric [15] compared the three major manufacturing sectors in China and proposed that the Chinese government should support market growth in a sector-neutral way, to make use of its huge domestic market, to better promote industrial upgrading. (4) Some scholars have studied the mechanism of TUMI. For example, Lin et al. [16] established a mechanism model of TUMI based on the partial least squares approach to structural equation modeling, and makes an empirical analysis using the panel data of G20. Seth and Alberto [17] systematically analyzed representative samples of primary products and light manufacturing industries in developing countries.

However, the existing research on TUMI is mainly through econometric methods and case studies to study the microenterprises and macroindustries. This ignores the dynamic cycle process of manufacturing transformation and lacks systematic analysis and evolution analysis of the relationship between influencing factors. To address these knowledge gaps, this paper assumes that the evolution of TUMI is a dynamic process, and various factors interact with each other, resulting in the emergence of complex phenomena. It can explore the role of different macro variables in the evolution process [18], and provide a new perspective for the research noted above. Therefore, taking the Guangdong–Hong Kong–Macau Greater Bay Area (GBA) as an example, this paper, first integrates relevant literature to construct the macro variable system of TUMI. Then, to explore the dynamic evolution process of different macro factors in the evolution process, an order parameter model was constructed. On this basis, the bottleneck factors in the process of GBA transformation can be found. This will help the government master TUMI law and better promote it.

The rest of the paper is arranged as follows: Section 2 summarizes the related literature. Section 3 puts forward the research method. Section 4 is experimental, including data sources and results. Section 5 discusses the theoretical and practical implications. Finally, the conclusion is presented.

## 2. Literature Review

### 2.1. The Concept of TMUI

The concept of TUMI is defined by the Ministry of Industry and Information Technology of China. It is pointed out in “Industrial Transformation and Upgrade Planning (2011–2015)” that TUMI aims to accelerate the transformation from traditional industrialization to new industrialization, and to promote the overall optimization and upgrading of industrial structure by changing the industrial development mode. However, the separation of these mechanisms is actually very difficult [19]. The proposal of Industry 4.0 [20] created more new development opportunities for traditional industries in transition [21]. Armando et al. [22] refined the enabling technologies, obstacles, and goals of industry through the management study of Industry 4.0. This helps managers of manufacturing enterprises in transition find the right technology enforcers for their goals. Sun et al. [23] established a digital manufacturing ecological framework based on Germany’s Industry 4.0. Therefore, this paper draws on the foresight and practical methods of Industry 4.0 to provide guidance for the factor selection of TUMI.

### 2.2. The Influence Factor of TMUI

Recently, many scholars have studied the factors that affect TUMI. On the aspect of industrial agglomeration, Fang et al. [24] found that it can promote rational allocation of industrial resources and reduce the cost and risk of manufacturing industry transformation and upgrading. On the aspect of the intelligent industry, Gereffi [25] found that TUMI fundamentally needs to develop to a higher technology stage. It can promote the manufacturing industry to engage in high value-added production [26]. On the aspect of technological innovation, Yang [27] found that it has a positive impact on the competitiveness of the manufacturing industry. Improving the technological innovation ability of the industry can break the technological barriers faced by the manufacturing industry [28]. On the aspect of market demand, Schumpeter emphasized the positive effect of market demand on innovation [29]. Romer et al. [30] found that the dual factors of technology and market are of great significance to TUMI. On the aspect of fixed asset, Wang et al. [31] found that fixed-asset investment leads to an imbalance of equipment demand in the manufacturing industry. This will affect the output structure of the industrial economy. William et al. [32] found that fixed-asset investment formed the means of production and improved industrial productivity. On the aspect of green development, Gramkow et al. [33] found that good green development measures can promote the development of TUMI. Zhou et al. [34] found that the traditional manufacturing industry leads to increasingly serious industrial pollution, and the environmental carrying capacity is facing severe challenges. This will restrict the development of TUMI.

It is worth noting that TUMI has always been a difficult task, involving various processes of regional industries [35] and often having to meet complex and diverse obstacles [36]. In a certain situation, the obstacles to the development of advanced manufacturing [37], the lack of infrastructure [38], and the negative attitude of the public towards green energy [39] may become the bottleneck of TUMI. Due to the complexity of TUMI, there are often different dominant bottlenecks in different stages of transformation. Therefore, this paper attempts to identify the bottleneck in the evolution process of TUMI at the present stage through the analysis method of synergetics, so as to provide a better decision-making basis for the government.

### 2.3. Existing Research Method

The literature described in Section 1 shows that existing scholars mainly use questionnaire surveys [7,10], case analyses [8,15,17], and regression models [9,14,16] to study the research on TUMI. However, these methods have some shortcomings for the study of TUMI. First, the questionnaire survey can go deep into the management personnel, employees, and relevant government personnel of the manufacturing industry to understand the situation faced in the process of TUMI [10]. However, these questionnaires cannot guarantee that respondents will answer the questions truthfully, nor can they capture a large amount of background information hidden behind the answers of the questionnaire. Second, the case analysis method can give an in-depth description and analysis of a certain enterprise or industry, but the case analysis in TUMI is often used at the micro level [40], and cannot be analyzed with regional economy as the research object. Third, econometric methods such as multiple regression are mature quantitative methods. However, most of the measurement methods used in current research on TUMI are static models, which ignore the dynamic cycle process of TUMI and lack systematic analysis and evolutionary analysis of the relationship between influencing factors.

Viewing TUMI from the perspective of synergetics, it is found that TUMI is a process of multisector coevolution and upgrading, in which the manufacturing industry constantly exchanges technology, resources, funds, information, and other elements with the outside world. Therefore, the order parameters method is very suitable for studying the evolution process of TUMI. The next section is devoted to the order parameters method.

### 2.4. The Order Parameters Method

The order parameter is one of the core concepts in synergetics theory. It refers to the macro parameter that plays a leading role in the process of the system from chaotic disorder state to cooperative order state. In the past, there are two methods to determine the order parameter. The first method is to determine the order parameters directly according to the understanding of the analysis object. For example, Meynhardt et al. [41] defined order parameters directly when analyzing the service ecosystem. In the analysis of high-tech virtual industrial clusters, Gao et al. [42] defines intellectual capital as an order parameter. This method of determining order parameters is based on the authors’ subjective understanding of system variables and requires rich and accurate experience and knowledge. The second method is to use the order parameter model to analyze several important variables to determine the order parameter. This method mathematically describes the evolution caused by the interaction between internal variables and external conditions of the system [43]. For example, Zhang et al. [44] found the power market operation efficiency by using the order parameter model. Zhang et al. [45] also used the order parameter model in the analysis of e-commerce systems. However, the optional range of sequence parameters is too limited, so the risk of losing important information is great.

Considering the characteristics of these two methods, TUMI is a nonlinear comprehensive process. In this paper, principal component analysis (PCA) is used to obtain the main features as candidate variables of order parameters. PCA can simplify the original complex problems and reflect the real situation as much as possible [46]. In this way, the order parameter can carry more information about the whole system.

## 3. Methodology

### 3.1. Data Source

In 2018, the outline of GBA development planning was issued, which indicated that it was necessary to build the strategic mission of developing an international advanced manufacturing base in the GBA. Therefore, this study mainly focused on TUMI in the GBA. The data used in this study were from the statistical yearbook of Guangdong Province from 2012 to 2019.

### 3.2. Variable Selection

**Figure 1:**
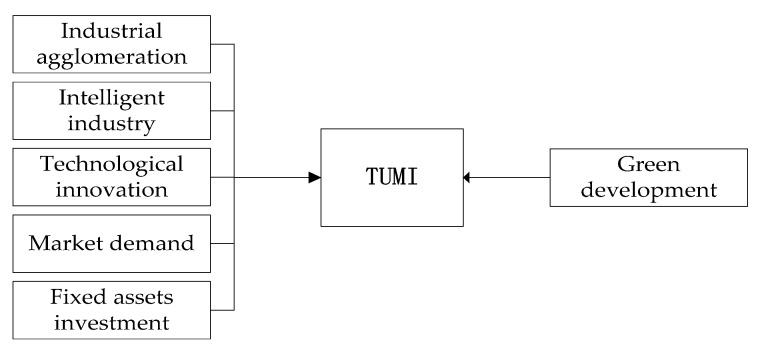
The macro factors of TUMI.

On the basis of the synergetic theory, referring to the government documents such as “GBA development plan”, six macro variables were selected to measure the TUMI process of the GBA, as shown in Figure 1 and Table 1.

Specifically, X1 plays an indispensable role in TUMI, which can effectively reduce the cost and risk of manufacturing development. TUMI must pay attention to the development of X2. Taking Tokyo Bay as an example, the traditional manufacturing industry is gradually replaced by a knowledge economy represented by the information industry. X3 is the core factor of TUMI. Through the improvement of industrial technology innovation ability, it is conducive to the sustainable transformation and upgrading of the industry. This is the inevitable choice to solve the deep-seated contradictions in the future development of the manufacturing industry. X4 for new products will stimulate enterprises to constantly innovate products and services. This can enable the company to obtain a sustainable competitive advantage, so as to achieve the purpose of industrial transformation and upgrading. X5 plays an important role in the process of TUMI, and can effectively provide corresponding supporting services. This can indirectly improve industrial productivity. Under environmental regulation, X6 has restricted TUMI. This makes the rigid restriction of the ecological environment become the restriction of manufacturing upgrading in GBA.

### 3.3. Research Method

To explore the dynamic evolution process of TUMI, we refer to the synergetic theory founded by German physicist Harken [47]. This theory shows that subsystems can change the system from a disordered state to an ordered state, or from an ordered state to a higher ordered state through the interaction of material, energy, and information exchange [48,49]. In recent years, synergetic theory, as one of the three emerging composite theories, has been widely used in the field of economy and management [50,51,52,53,54,55,56,57,58]. Given the mutual influence between the variables of the TUMI family, this paper considers that TUMI is a dynamic feedback cycle process. The synergetic order parameter model can effectively capture the evolution trajectory characteristics of TUMI and reveal its internal dynamic process. In this section, we discuss the PCA-based order parameter model in detail.

First, assume that P index variables are $V=\left(U_{1},U_{2},\ldots ,U_{p}\right)$. B represents a number of common factors with the same explanation, that is, two orthogonal. The formula of factor analysis follows:

(1) $$\{\begin{array}{c} B_{1}=a_{11}U_{1}+a_{12}U_{2}+\cdots +a_{1p}U_{p}\\ {\;B}_{2}=a_{21}U_{1}+a_{22}U_{2}+\cdots +a_{2p}U_{p}\\ \cdots \\ {\;B}_{q}=a_{q1}U_{1}+a_{q2}U_{2}+\cdots +a_{qp}U_{p} \end{array}$$

where $V={\left(U_{1},U_{2},\ldots ,U_{p}\right)}^{T}$, $a_{i,j}\left(i=1,\ldots ,p;j=1,\ldots ,q\right)$ is the load of the variable on factor i and factor j. When i≠j, B is the principal component variable, which is represented by the time series as $B={\left(B_{1},B_{2},\ldots ,B_{q}\right)}^{T}$.

PCA reduces the dimension of multivariate data and obtains new independent variables. In this paper, principal component variables are used as candidate variables of order parameters. This method needs to satisfy the following assumptions:

(1) There is a certain correlation between variables, which is the premise of PCA.
(2) This method has Markov property. In other words, the future state only depends on the current state and has nothing to do with the past.
(3) The principal component variable has the effect of dependence or depression, which is affected by the change rate of the dependent variable.
(4) When all principal component variables are 0, the effect of independent variables on dependent variables disappears.

Second, according to the Markov principle, the principal component variables are expressed by difference equations and nonlinear differential equations. The formula follows:

(2) $$B_{j}\left(k+1\right)={\tilde{f}}_{j}\left(B_{1}\left(k\right),B_{2}\left(k\right),\cdots ,B_{q}\left(k\right)\right)$$

(3) $${\dot{B}}_{j}\left(t\right)=f_{j}\left(B_{1}\left(t\right),B_{2}\left(t\right),\cdots ,B_{q}\left(t\right)\right)$$

where k=1, 2,…,m−1, $\tilde{f}$ is a nonlinear mapping, and f is a nonlinear function. Since the principal component variables are not correlated, $f_{j}$ has the following form:

(4) $$f_{j}\left(B_{1}\left(t\right),B_{2}\left(t\right),\cdots ,B_{q}\left(t\right)\right)=\left[\gamma_{j}+g_{j}\left(B_{1}\left(t\right),B_{2}\left(t\right),\cdots ,B_{q}\left(t\right)\right)\right]B_{j}\left(t\right)$$

$\gamma_{j}$ is the inherent rate of change of $B_{j}\left(t\right)$ and is the damping coefficient of $B_{j}\left(t\right)$. At the same time, it satisfies $\gamma_{j}\neq 0$. $g_{j}\left(B_{1}\left(t\right),B_{2}\left(t\right),\;\ldots ,B_{q}\left(t\right)\right)$ is the characterization function of the interaction of each principal component variable with $B_{j}\left(t\right)$. When all principal component variables are 0, there is $g_{j}\left(0,0,\ldots ,0\right)=0$, which means that the influence of independent variables on dependent variables is 0.

The linear term can be separated from the right end of Equation (3). The order parameter model can be expressed by an autonomous differential equation:

(5) $${\dot{B}}_{j}\left(t\right)=f_{j}\left(B_{1},B_{2},\cdots ,B_{q}\right)=\gamma_{j}B_{j}+g_{j}\left(B_{1},B_{2},\cdots ,B_{q}\right)B_{j}$$

Let ${\dot{B}}_{j}\left(t\right)=0$; obviously the origin $B^{*}{}_{j}\left(t\right)=\left(0,0,\ldots ,0\right)$ is the equilibrium point.

Then, the linearization equation of differential Equation (5) in $B^{*}{}_{j}\left(t\right)$ is derived. The partial derivative matrix follows:

(6) $$DF=\left[\begin{array}{cccc} \frac{\partial f_{1}}{\partial z_{1}} & \frac{\partial f_{1}}{\partial z_{2}} & \ldots & \frac{\partial f_{1}}{\partial z_{q}}\\ \frac{\partial f_{2}}{\partial z_{1}} & \frac{\partial f_{2}}{\partial z_{2}} & \ldots & \frac{\partial f_{2}}{\partial z_{q}}\\ \ldots & \ldots & \ldots & \ldots \\ \frac{\partial f_{q}}{\partial z_{1}} & \frac{\partial f_{q}}{\partial z_{2}} & \ldots & \frac{\partial f_{q}}{\partial z_{q}} \end{array}\right]$$

where $\frac{\partial f_{j}}{\partial z_{j}}=\gamma_{j}+g_{j}\left(B_{1},\ldots ,B_{q}\right)+B_{j}\frac{\partial g_{j}\left(B_{1},\ldots ,B_{q}\right)}{\partial z_{j}}$, $\frac{\partial f_{j}}{\partial z_{i}}=B_{j}\frac{\partial g_{j}\left(B_{1},\ldots ,B_{q}\right)}{\partial z_{i}}$.

Combined with $g_{j}\left(0,0,\ldots ,0\right)=0$, at the equilibrium point $B^{*}=\left(0,0,\ldots ,0\right)$, the partial derivative matrix is:

(7) $$DF\left(B^{*}\right)=\left[\begin{array}{cccc} \gamma_{1} & 0 & \ldots & 0\\ 0 & \gamma_{2} & \ldots & 0\\ \ldots & \ldots & \ldots & \ldots \\ 0 & 0 & \ldots & \gamma_{q} \end{array}\right]$$

The linearization equation ${\left({\dot{B}}_{1},{\dot{B}}_{2},\ldots ,{\dot{B}}_{q}\right)}^{T}=DF\left(\dot{B}\right){\left(B_{1},B_{2},\ldots ,B_{q}\right)}^{T}$ corresponding to the origin of Equation (4) can be obtained, that is

(8) $${\dot{B}}_{j}=\gamma_{j}B_{j}$$

Equation (8) is the eigenvalue of matrix $DF\left(B^{*}\right)$, where $\lambda_{i}=\gamma_{i}\neq 0$. Since the nonlinearity weakens near the equilibrium point, it is necessary to judge whether or not EquationFormula (4) is stable at the origin.

Combined with the Lyapunov stability criterion, when $\gamma_{j}<0$, $B_{j}\left(t\right)$ corresponding to small damping coefficient $\gamma_{j}$ is a slow variable. The large damping coefficient is a fast variable, which keeps the stability of Equation (4) at the origin. When $\gamma_{j}>0$, $B_{j}\left(t\right)$ is a slow and unstable variable, while other variables are fast, so Equation (5) is unstable. It is worth noting that the model is dominated by slow variables at this moment, and the slow variables are transformed into order parameters.

According to this method, the variables are divided into two groups: fast variable, that is: $B_{l}\left(t\right),l=1,\;2,\;\ldots ,\;h$; and slow variable, that is: $B_{s}\left(t\right),s=h+1,h+2,\ldots ,q$.

Because the principal component variables are not correlated, $g_{j}\left(B_{1},B_{2},\cdots ,B_{q}\right)=0$. It has an effect on the self change rate of the dependent variable $B_{s}$. It can be recorded as the action function $g_{ji}\left(B_{i}\right)$ of $B_{i}$ to the change rate of $B_{j}$.

We record its influence on the rate of change of the dependent variable $B_{s}$ as the function of $B_{i}$ on the rate of change of $B_{j}$, that is, $g_{ji}\left(B_{i}\right)$. Therefore, we can deduce that:

(9) $$g_{j}\left(B_{1},B_{2},\cdots ,B_{q}\right)=\overset{q}{\underset{i=1}{\sum }}g_{ji}\left(B_{i}\right)$$

According to the synergetic principle, when the fast variable is represented by the slow variable, the adiabatic approximation can be used to deduce the following equation:

(10) $$B_{s}\left(t\right)=-g_{ss}^{-1}\left(\gamma_{s}+\overset{q}{\underset{i=1,i\neq s}{\sum }}g_{si}\left(B_{i}\left(t\right)\right)\right)$$

The solution of a fast variable dependent on a slow variable can be obtained simultaneously:

(11) $$B_{s}\left(t\right)=B_{s}\left(B_{1}\left(t\right),B_{2}\left(t\right),\ldots ,B_{h}\left(t\right)\right)$$

Thus, the order parameter equation can be obtained:

(12) $${\dot{B}}_{l}=\gamma_{l}B_{l}+g_{l}\left(B_{l},B_{s}\left(B_{l}\right)\right)B_{l}$$

Equation (12) represents the evolution trajectory of order parameters, which can effectively observe the operation path of order parameters.

## 4. Empirical Analysis

### 4.1. Data Preprocessing

Because the dimension of each evaluation index is different, it needs to be summarized by the entropy algorithm. The formula follows:

(13) $$W_{i}=\frac{\left(1-e_{j}\right)}{\sum_{j=1}^{n}\left(1-e_{j}\right)}$$

where $e_{j}=-k\sum_{j=1}^{m}\left(Y_{ij}-\ln Y_{ij}\right)$, $Y_{ij}=\frac{{X^{\prime }}_{ij}}{\sum_{i=1}^{m}{X^{\prime }}_{ij}}$, m is the number of years, and *n* is the number of evaluation indicators. The index data from 2011 to 2018 are shown in Table 2.

### 4.2. Results and Analysis

The result of KMO is 0.713, indicating that the extracted principal component variables have a strong correlation. Bartlett’s test showed that the value of sig is equal to 0, indicating that it has good significance.

The first principal component can explain 84.976% of the original variance, and the second principal component can explain 11.944% of the original variance. Combined with Table 3, we can see that the first principal component contains U_1_–U_5_, which can be called the overall development coefficient of the industry. The second principal component is only U_6_, which can be called the environmental factor.

The formula follows:

(14) $$B_{1}=0.19U_{1}+0.19U_{2}+0.19U_{3}+0.19U_{4}+0.18U_{5}-0.12U_{6}$$

(15) $$B_{2}=0.07U_{1}+0.22U_{2}+0.18U_{3}+0.24U_{4}-0.04U_{5}+1.12U_{6}$$

By calculating the scores of B1 and B2 from 2011 to 2018 (Table 4), we can intuitively understand the overall development and green development trend of the manufacturing industry in GBA. Intuitively, from 2011 to 2018, the overall development of the manufacturing industry in nine cities showed an increasing trend year by year, while the change of green development showed a wave dynamic trend. Therefore, it is necessary to further calculate the order parameters of B1 and B2, which can effectively observe the trajectory of B1 and B2 interaction.

Due to the large difference between the positive and negative scores of principal components from 2011 to 2018, in order to reduce the influence on the operation of order parameters, the scores are smoothed. According to this analysis, the fitting difference equation is obtained:

(16) $$B_{1}\left(k+1\right)=1.71B_{1}\left(k\right)+0.05B_{1}\left(k\right)B_{2}\left(k\right)-0.31B_{1}{}^{2}\left(k\right)$$

(17) $$B_{2}\left(k+1\right)=0.71B_{2}\left(k\right)-0.11B_{1}\left(k\right)B_{2}\left(k\right)-0.04B_{2}{}^{2}\left(k\right)$$

After continuous treatment, the differential equation follows:

(18) $${\dot{B}}_{1}=0.71B_{1}+0.05B_{1}B_{2}-0.31B_{1}{}^{2}$$

(19) $${\dot{B}}_{2}=-0.29B_{2}-0.11B_{1}B_{2}-0.04B_{2}{}^{2}$$

where the linear coefficients $B_{1}$ and $B_{2}$ represent the self growth rate of variables, $B_{1}$ has self growth effect, $B_{2}$ has self attenuation effect.

The interaction term $B_{1}B_{2}$ indicates the interaction between $B_{1}$ and $B_{2}$. The positive interaction term of Formula (18) indicates that $B_{2}$ has a positive effect on $B_{1}$, and the negative interaction term of Formula (19) indicates that $B_{1}$ has a negative effect on $B_{2}$. The square term represents the influence of the coefficient with the change of the variable itself. The negative square term in Formulas (18) and (19) indicates that $B_{1}$ and $B_{2}$ have a certain degree of blocking effect on their own development over time.

The slow variable $B_{1}$ has become an order parameter in the current state, which is composed of U_1_–U_5_. According to Equation (14), it can be found that the weight of the above indicators is equal, which is in line with the basic situation of TUMI in GBA. In practice, GBA has already become an important manufacturing base in the world, with a large-scale, high-level, and prominent manufacturing industry system. In recent years, TUMI in GBA mainly focuses on the development of the advanced manufacturing industry, the investment of scientific and technological resources, the comprehensive integration of new product R&D, and sales. The advanced manufacturing industry, represented by new product consumption and R&D technology, is gradually replacing the traditional manufacturing industry. Therefore, at the current stage, GBA forms its core technology, constructs a high-end manufacturing industry chain, and realizes the balanced development of the manufacturing industry.

**Figure 2:**
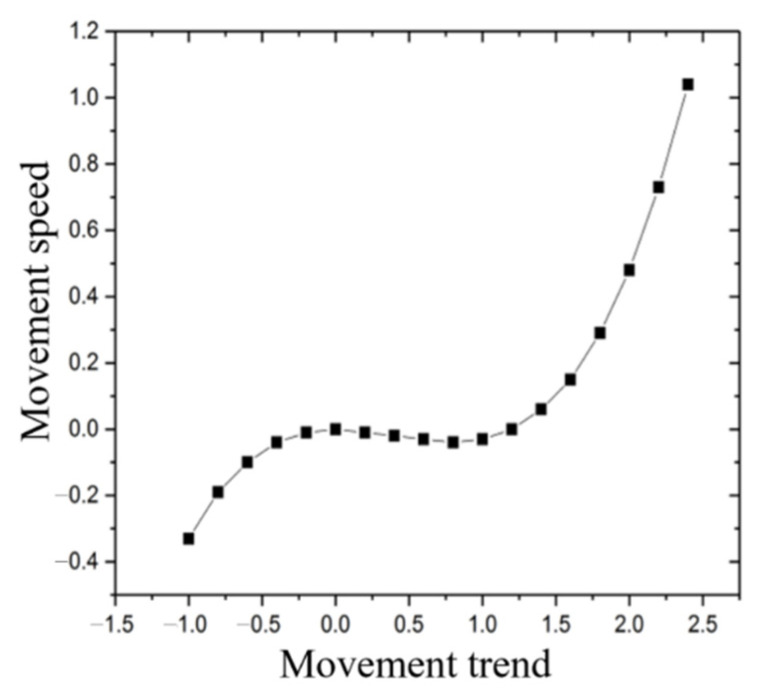
The potential function simulation diagram.

Second, according to the adiabatic approximation principle, in the case of $B_{2}\neq 0$, ${\dot{B}}_{2}=0$ is ordered, and the fast variable $B_{2}$ is represented by the slow variable $B_{1}$ as $B_{2}=-7.25-2.75B_{1}$. After substituting it into Formula (18), the function $C\left(B_{1}\right)$ can be introduced to satisfy $-\frac{\partial C}{\partial B}=-\frac{dB_{1}}{dt}$. Thus the potential function formula $C\left(B_{1}\right)=-0.18B_{1}{}^{2}+0.15B_{1}{}^{3}$ can be derived. Through the simulation of the potential function, the trajectory of $B_{1}$’s virtual particle is obtained, as shown in Figure 2. The order parameter breaks away from the original attraction point and forms a dissipative state in the system.

The growth rate of the order parameter first increases and then decreases with time, and finally tends to a stable nonzero state. In other words, the current development model will gradually get into trouble with the hindrance and need to break the balance through new order parameters. That is, in the process of the GBA manufacturing industry to high-end TUMI, the continuous improvement and improvement of the overall development index promoted TUMI to enter a period of rapid development. However, with the continuous improvement of the overall development level of the manufacturing industry, the blocking effect of industrial pollution on TUMI will gradually become prominent. This has a crowding-out effect on the contribution of the overall development indicators to TUMI. At the same time, environmental problems become the bottleneck of its transformation. Therefore, in the late stage of transformation and upgrading, the government pays attention to the coordinated development of the overall development indicators. They should seize the opportunity to shift the focus of TUMI to the green development of the manufacturing industry, to make the six factors develop in coordination, so as to ensure a new round of TUMI development.

## 5. Discussion

The study found that the weights of U_1_–U_5_ indexes were basically flat, which was in line with the basic situation of TUMI in GBA. In practice, it shows the mutual promotion of these factors. Taking the technical index (U_3_) as an example, through the introduction of advanced technology, it can effectively influence other indicators. The application of fault diagnosis technology [59] can enhance the intelligence of the manufacturing industry (U_2_). The application of public opinion analysis technology [60] can help enterprises understand the market situation, to improve the market demand of the manufacturing industry (U_4_). Gao et al. [61] found that under the influence of industrial association, industrial agglomeration (U_1_) has a promoting effect on technological progress.

### 5.1. Theoretical Implication

The high-quality development of TUMI has become a hot research topic, but the related research focuses on other aspects, and there is little research on the development of TUMI from the perspective of synergetics theory.

First, this paper enriches the literature on the transformation and upgrading of the regional manufacturing industry. Applying synergetics theory to the transformation and upgrading of the manufacturing industry can provide an innovative perspective. It can provide guidance for TUMI.

Second, based on the order parameter model, this paper makes a comprehensive study on the variables that affect TUMI. This method is different from the existing case analysis and empirical analysis methods to measure macro variables, which can effectively distinguish the factors of regional TUMI. It is worth noting that it proves the important role of environmental rules.

Third, in the selection of order parameter candidate variables, this paper creatively uses principal component analysis to select candidate variables. It has a certain reference value for the selection method of the order parameter.

### 5.2. Practical Implication

From a practical point of view, the insights provided in this study can be used to formulate reasonable measures and provide scientific recommendations for regional governments, specifically:

First, the overall industrial development factors are composed of U_1_–U_5_, which jointly promote the overall transformation and upgrading of the regional manufacturing industry. However, it is worth noting that these factors are not simply parallel, but need to develop in synergy with each other. Among them, U_1_ is the main factor, which gathers industrial resources and provides development space. U_2_ is the ontological factor, improving the production process and climbing the high end of the value chain. U_3_ is the core factor, which improves the technological content of the manufacturing industry and saves the investment of resources. U_4_ is the traction factor, which updates the product form and drives the industry iteration. U_5_ plays a supporting role, making up for market failure and promoting the coordinated development of multiple subjects. To summarize, the coordinated development of the five factors contribute to the overall development of the industry.

Second, although U_1_–U_5_ is an important power source for GBA transformation and upgrading, U_6_ is an important coordination mechanism to break the bottleneck. The environmental pollution caused by industrial development will limit further TUMI, or even bring the transformation and upgrading to a standstill. At this time, environmental problems become the bottleneck of transformation and upgrading. Therefore, the government should attach importance to U_6_ in due course. For example, the government’s environmental protection policy can activate the green consciousness of enterprises, saving the environmental protection cost of enterprises and energy consumption. This can promote the development of the external path of green development. At the same time, green development provides the possibility for further development of the industry. The overall development factors of the industry and green development factors need to be coordinated to promote the smooth completion of TUMI in GBA.

## 6. Conclusions

This study aims to analyze the development trend of TUMI in GBA (2008–2018). On the basis of synergetics, the order parameter method of factor analysis is used to analyze these factors. The results show that:

(1) There are five slow variable factors, such as intelligent manufacturing industry, technological innovation, scale agglomeration, market demand, and fixed asset investment, which are important power sources of TUMI in GBA. The development of these factors is relatively mature, and they cooperate with each other. However, it is worth noting that these factors are not a simple parallel relationship, but a coordinated development. Therefore, at the present stage, GBA should form an independent core technology through macroeconomic regulation and rational allocation of social resources. Through the construction of a high-end manufacturing industry chain, the intelligent development of the manufacturing industry is realized and the balanced development of the manufacturing industry on the path of transformation and upgrading is promoted.

(2) With the continuous development of manufacturing industry, its environmental pollution will limit the development of TUMI. Like a fast variable of manufacturing development ecology, green development is an important coordinating factor in removing bottlenecks. The implementation of environmental management and control systems for manufacturing enterprises makes the rigid constraint of the ecological environment become the driving force for upgrading the manufacturing industry. The green development factor, together with the other five factors, promotes the transformation and upgrading, and its effect is far higher than that of independent development.

The survey data come from a first tier urban agglomeration in southern China, whose characteristics may be quite different from those of other urban agglomerations. The geographical location and economic development obviously have an important impact on the transformation and upgrading of regional manufacturing industry. Although this study is limited to a specific region, through further research this knowledge can be globally adopted and verified. Most situations are common in the development of the global industry. It is also very useful for the manufacturing cluster in other regions. The advantage of this research method is that it can directly analyze the structure of order parameters, grasp the composition of order parameters, and retain the maximum amount of information. However, this method is most effective when the correlation coefficient between indicators is large.

## Figures and Tables

**Table 1:** The macro factors for TUMI.

Hidden Layer Variables	Explanatory Variables
Industrial agglomeration (U1)	Gross industrial output value above scale (X1)
	Industrial added value above scale (X2)
Intelligent industry (U2)	Value added of advanced manufacturing industry (X3)
	Value added of high-tech manufacturing industry (X4)
Technological innovation (U3)	R&D personnel (X5)
	Internal expenditure of R&D funds (X6)
Market demand (U4)	Output value of new products (X7)
	Revenue from sales of new products (X8)
Fixed assets investment (U5)	Fixed assets investment in manufacturing industry (X9)
Green development (U6)	Discharge quantity of industrial wastewater (X10)
	Industrial exhaust gas emission quantity (X11)
	Quantity of industrial solid waste (X12)

**Table 2:** The index data of 9 cities.

	*U*_1_	*U*_2_	*U*_3_	*U*_4_	*U*_5_	*U*_6_
2011	0.00	0.00	0.00	0.00	0.00	0.59
2012	0.03	0.07	0.14	0.04	0.15	1.00
2013	0.31	0.28	0.27	0.14	0.20	0.80
2014	0.47	0.36	0.39	0.23	0.29	0.85
2015	0.58	0.46	0.51	0.34	0.67	0.00
2016	0.75	0.62	0.64	0.60	0.87	0.12
2017	0.83	0.82	0.79	0.84	1.00	0.73
2018	1.00	1.00	1.00	1.00	0.75	0.28

**Table 3:** The principal component variable component matrix.

	*U*_1_	*U*_2_	*U*_3_	*U*_4_	*U*_5_	*U*_6_
Component 1	0.988	0.983	0.986	0.971	0.942	−0.596
Component 2	0.053	0.159	0.129	0.173	−0.028	0.801

**Table 4:** The principal component scores in GBA.

Year	2011	2012	2013	2014	2015	2016	2017	2018
B1	−1.24	−1.14	−0.66	−0.39	0.30	0.75	0.99	1.40
B2	−0.71	0.67	0.40	0.72	−1.67	−0.95	1.26	0.28

## Data Availability

The data in this paper are from the statistics bureau of Guangdong province. http://stats.gd.gov.cn/ (accessed on 12 February 2021).
